# Reducing Suicidal Ideation: Cost-Effectiveness Analysis of a Randomized Controlled Trial of Unguided Web-Based Self-help

**DOI:** 10.2196/jmir.1966

**Published:** 2012-10-26

**Authors:** Bregje A.J van Spijker, M. Cristina Majo, Filip Smit, Annemieke van Straten, Ad J.F.M Kerkhof

**Affiliations:** ^1^Department of Clinical Psychology and the EMGO+ Institute for Health and Care ResearchFaculty of Psychology and EducationVU University AmsterdamAmsterdamNetherlands; ^2^Trimbos InstituteNetherlands Institute of Mental Health and AddictionUtrechtNetherlands; ^3^Department of Epidemiology and BiostatisticsEMGO+ Institute for Health and Care ResearchVU University Medical CentreAmsterdamNetherlands

**Keywords:** suicidal ideation, randomized controlled trial, cost-effectiveness, Internet, cognitive behavior therapy

## Abstract

**Background:**

Suicidal ideation is highly prevalent, but often remains untreated. The Internet can be used to provide accessible interventions.

**Objective:**

To evaluate the cost-effectiveness of an online, unguided, self-help intervention for reducing suicidal ideation.

**Methods:**

A total of 236 adults with mild to moderate suicidal thoughts, defined as scores between 1-26 on the Beck Scale for Suicide Ideation (BSS), were recruited in the general population and randomized to the intervention (n = 116) or to a waitlist, information-only, control group (n = 120). The intervention aimed to decrease the frequency and intensity of suicidal ideation and consisted of 6 modules based on cognitive behavioral techniques. Participants in both groups had unrestricted access to care as usual. Assessments took place at baseline and 6 weeks later (post-test). All questionnaires were self-report and administered via the Internet. Treatment response was defined as a clinically significant decrease in suicidal ideation on the BSS. Total per-participant costs encompassed costs of health service uptake, participants’ out-of-pocket expenses, costs stemming from production losses, and intervention costs. These were expressed in Euros (€) for the reference year 2009.

**Results:**

At post-test, treatment response was 35.3% and 20.8% in the experimental and control conditions, respectively. The incremental effectiveness was 0.35 − 0.21 = 0.15 (SE 0.06, *P *= .01). The annualized incremental costs were −€5039 per participant. Therefore, the mean incremental cost-effectiveness ratio (ICER) was estimated to be −€5039/0.15 = −€34,727 after rounding (US −$41,325) for an additional treatment response, indicating annual cost savings per treatment responder.

**Conclusions:**

This is the first trial to indicate that online self-help to reduce suicidal ideation is feasible, effective, and cost saving. Limitations included reliance on self-report and a short timeframe (6 weeks). Therefore, replication with a longer follow-up period is recommended.

## Introduction

Suicidal ideation is highly prevalent and causes considerable disease burden [1], but often remains untreated [2]. Frequently reported barriers to seeking help include a preference to handle the problem alone, believing the problem is not severe, and believing treatment will not be effective [2]. For these reasons, an online self-help intervention specifically aimed at reducing suicidal ideation was developed [3]. Self-help can be defined as a standardized psychological treatment that a participant can work through independently. The rationale for using online delivery for this intervention included the reach, accessibility, and anonymity of the Web, thereby facilitating dissemination. Web-based interventions have been found effective for a range of mental disorders (eg, depression, anxiety, and problem drinking) [4-7].

The potential economic advantages of Web-based interventions are among commonly cited motivations for their development [8]. Indeed, promising results have been published for Web-based interventions targeting both somatic [9-10] and psychological problems [11-15]. However, being a relatively young research field, economic evaluations of Web-based interventions are still scarce and often have limitations [8].

For face-to-face psychological treatments targeting suicidality, some empirical evidence is available for cognitive behavior therapy (CBT) [16-17], dialectical behavior therapy (DBT) [18-20], problem-solving treatment (PST) [21-22], and mindfulness-based cognitive therapy (MBCT) [23-25]. Economic evaluations of psychological treatments for suicidality are almost non-existent, which has been attributed to a general lack of unambiguous effectiveness of treatment programs [26-27]. One study comparing manual-assisted cognitive behavior therapy (MACT) with care as usual found indications that MACT was valuable from an economic perspective; however, results were not conclusive [28]. Furthermore, a review of therapies for borderline personality disorder indicated that DBT could potentially be cost-effective [29].

The economic impact on society of not taking preventive measures (ie, the costs of suicide) have been estimated to be well over £1,000,000 per suicide (2005 prices) [27]. Although no similar estimates have been reported regarding suicidal ideation, these are likely to be substantial when considering the economic burden of depression [30-31], a common mental disorder in people with suicidal ideation.

This paper reports the results of an economic evaluation of a randomized controlled trial comparing online self-help for suicidal ideation with a waitlist control condition (Netherlands Trial Register, NTR1689).

## Methods

### Design and Participants

Participants were recruited between October 2009 and November 2010 from the Dutch general population by means of advertisements in newspapers, relevant websites, and Google AdWords. The methods used in this trial have been described in detail elsewhere [3]. To be included, people had to be over 18 years, have access to the Internet and a valid email address, and have a good command of the Dutch language. In addition, they needed to present with a score between 1 and 26 on the Beck Scale for Suicide Ideation (BSS) [32] suggesting mild to moderate suicidal ideation, and a score < 40 on the Beck Depression Inventory (BDI) [33], to avoid including people with severe levels of depression. These criteria were established in consultation with clinical experts.

**Figure 1:**
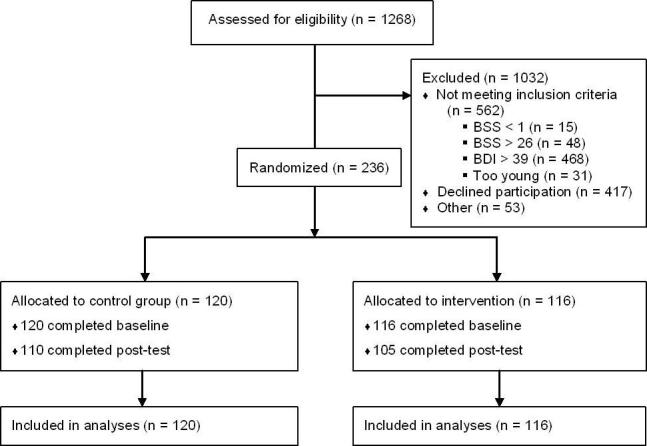
Flowchart of participants in the trial.

Eligibility was assessed using an online application procedure. In total, 1268 respondents filled in the screening questionnaires (BSS and BDI). Respondents who exceeded the cutoff scores (562/1268, 44.32%) were referred to other (mental health) services by means of an automated response. Eligible respondents were requested to fill in their email address, after which an information brochure and an informed consent form were emailed to them. A small number did not fill in their email address and were consequently excluded (53/1268, 4.18%). After returning the informed consent form, on which participants had to disclose their identity and that of their family physician, participants (n = 236) were stratified for gender and randomized in blocks of 20 to the intervention (n = 116) or to the waitlisted information-only control condition (n = 120) by an independent researcher using random allocation software. Most of the eligible respondents (417/706, 59.1%) did not return their informed consent form, possibly due to the lack of anonymity when participating (see Figure 1).

After randomization, participants in the intervention group received log-in codes for the self-help intervention. Participants in the control group received a link to an information website and were informed that they would receive access codes to the intervention 6 weeks later. It is worth noting that all participants, in both conditions, had unrestricted access to care as usual (CAU) and were encouraged to make use of this. In the Netherlands, people most commonly go to a family physician who can refer to specialized mental health services if necessary.

Because this study was conducted in a vulnerable population, safety procedures were employed. Each time a participant in either condition exceeded cutoff scores on suicidal ideation or depressive symptoms, a risk assessment was carried out over the phone. If necessary, or if a participant could not be reached, their family physician was contacted [3]. The study was approved by the Medical Ethics Committee of the VU University Medical Centre (registration number 2008/204).

### Intervention

The experimental group received an online, unguided, self-help intervention aimed at decreasing the frequency and intensity of their suicidal ideation. This intervention is based on CBT (DBT, PST, and MBCT). All of these treatment programs have evidence for their effectiveness in reducing suicidality [18,24,34-35].

The intervention consists of six weekly modules which consecutively focus on (1) the repetitive character of suicidal thoughts [36], (2) dealing with intense emotions, (3) identifying negative automatic thoughts, (4) learning to recognize thinking patterns, (5) reformulating negative thoughts, and (6) relapse prevention. A more detailed description of the intervention has been previously published [3]. The intervention is currently available through 113Online (www.113online.nl), a Dutch online suicide prevention platform [37,38].

Participants were encouraged to follow one module per week and they received an automated weekly motivating email. Approximately half (56.0%, 65/116) of the participants in the intervention group completed at least three modules of the intervention, 21.6% (25/116) completed the whole intervention, and a similar percentage (22.4%, 26/116) did not start the intervention.

If desired, participants were able to ask questions pertaining to the intervention via the website. Questions asked were often about specific exercises (eg, “What thoughts should I tally?” and “Should I continue worry time in the second module?”) or about the other aspects of the website (eg, “How long will my log-in codes be valid for?”). These were answered by the researchers, taking an average of 6 minutes per participant over the entire intervention period.

The control group received a link to a website created for the study that provided information on suicidality, such as prevalence, warning signs, and risk factors. Pretesting indicated that a maximum of 15 minutes was needed to read this information. In addition, links to relevant mental health centers were provided and participants were advised to use these.

### Power Analyses

Sample size was based on the expected effect on the primary outcome measure (ie, the reduction of suicidal thoughts). In order to be able to detect an effect size of 0.35 with alpha = .05 and beta = .80, 100 subjects were needed in each condition. Including an expected drop-out attrition rate of 20% to 30% in each group, the sample size was determined at 260.

### Outcome Measures

Questionnaires were self-report and administered via the Internet. For the current paper, data from baseline and post-test (6 weeks after baseline) were used.

#### Primary Clinical Outcome

The primary clinical outcome in this paper is suicidal ideation, assessed using the BSS [32]. This self-report questionnaire consisted of 21 items, each scored on a 0-2 scale. The total score was obtained by adding items 1-19 (range 0-38). The last two items relate to suicide attempts and the intent to die during the most recent attempt. Internal reliability of the BSS is high, with Cronbach alpha ranging between .87 and .97 [39]. In this study, Cronbach alpha = .89 at baseline.

#### Resource Use and Costing

A societal perspective was adopted in this study; therefore, the costs of health service uptake, patients’ out-of-pocket costs, and production losses in paid work were included. Data on health care uptake and production losses were collected using the Trimbos/Institute of Medical Technology Assessment Questionnaire for Costs associated with Psychiatric Illness (TIC-P) [40], a health service receipt questionnaire that is widely used in economic evaluations in the Netherlands. This produces three cost categories: direct medical costs, direct non-medical costs, and indirect non-medical costs. Data were collected for two periods: the 6 weeks prior to baseline and the 6 weeks following baseline.

Direct medical costs relate to the utilization of health care services. To calculate these costs, health service units were multiplied by their standard full economic cost prices as reported in the Dutch guidelines [41] for health economic evaluations for the reference year 2009 (see Table 1). The costs of prescription psychotropic drugs (eg, antidepressants, benzodiazepines, and antipsychotics) were calculated as the price per standard daily dose as reported in the Dutch Pharmacotherapeutic Compass [42], multiplied by the number of prescription days, plus pharmacists’ dispensing costs of €14 per prescription.

**Table 1:** Direct medical and direct non-medical costs by health service type.

Health service type	Direct medical costs		Direct non-medical costs		
	Unit	Unit cost price^a ^(€)	Distance (km)^b^	Time (h)^b^	Unit cost price^c ^(€)
General practitioner	Contact	28	1.1	1	15.72
Company doctor^d^	Contact	28	17.6	0.5	9.77
Social worker	Contact	65	5	2	29
Private practice psychotherapist, psychiatrist	Contact	90^e^	7	2	29.40
Alcohol and drug consultant (CAD)	Contact	171	10^f^	3	42.50
Regional mental health service	Contact	171	7	3	41.90
Physiotherapist	Contact	36	2.2	2	28.44
Mental hospital	Contact	173	7	4	54.40
Medical specialist general hospital	Contact	72	7	3	41.90
Alternative treatment^g^	Contact	50.70	5	1	16.50
Daycare, mental health treatment	Contact	154	7	4	54.40
Home care	Hour	35	NA	NA	NA
Informal care (family, friends)^h^	Hour	12.50	NA	NA	NA

^a ^Integral unit cost prices [41] presented in 2009 €.

^b ^Based on average distances (in special tariff taxi and public transport zones) and travel + waiting + treatment times (in hours) for receiving treatment [41].

^c ^Costs = (0.2 × km) + 3 + (12.5 × hrs). With €0.20 = cost per km; €3 = 1 h parking time; €12.5 = 1 h time [41].

^d ^No parking costs assumed.

^e ^Own calculation, valued as average of private practice psychotherapist and psychiatrist [41].

^f ^Assumed as CAD were more dispersed than regional mental health services.

^g ^Own calculation, valued as average of homoeopath and acupuncturist [41].

^h ^Valued as domestic help [41].

Direct non-medical costs encompassed participants’ travel expenses to receive professional help and loss of leisure time, the latter valued at €12.50 per hour [41]. Additionally, informal caregivers’ (ie, friends, neighbors, and family) use of time (eg, running errands for participants), was valued at €12.50 per hour (see Table 1).

Finally, the costs stemming from production losses in paid work (indirect non-medical costs) were calculated from the number of days absent from work (absenteeism), plus the number of days spent at work with reduced efficiency, corrected for degree of inefficiency (presenteeism). Table 2 reports the age-specific economic costs of each hour of lost productivity for men and women.

**Table 2:** Productivity costs by gender and age class [41].

Age range	Gender	
	Men (€)^a^	Women (€)^a^
15-19	9.65	8.76
20-24	17.75	17.18
25-29	24.19	23.62
30-34	29.65	27.54
35-39	34.03	29.25
40-44	36.67	29.06
45-49	38.32	28.91
50-54	39.06	29.25
55-59	39.38	29.50
60-64	39.13	28.67
65+	39.13	28.67
		

^a ^Costs are indexed from the Collective Labor Agreement, 2008 (2.8%) and presented in 2009 € values.

The total associated costs (in Euros) can be converted to US dollars using the purchasing power parity (PPP) exchange rates reported by the Organisation for Economic Co-operation and Development (OECD) [43], which converts currency while taking into account the differential buying power across countries. For the reference year 2009, US $1.00 was equivalent to €0.848173.

#### Per-participant Intervention Costs

In estimating per-participant intervention costs, the average time spent on the intervention was valued at €12.50 per hour (leisure time value [41]) for an average of 10.5 hours per participant over the 6-week intervention period. In addition, psychologist time spent answering questions was included for an average of 6 minutes per participant over the course of the intervention (at €154/hour [41]). Further costs were related to website maintenance, which amounted to €1740 and €10,000 per annum for upgrading and hosting the website, respectively.

Relying on data from a Dutch population survey on suicidality [44] and Statistics Netherlands [45], 91% of the Dutch adult population with suicidal ideation (N = 180,000) has Internet access (n = 163,800). Taking a conservative approach, it was assumed that 40% would search for online help, and 10% of these people would engage in the online self-help intervention. This resulted in an estimated usage by 6552 participants per year.

Based on the above assumptions and data, the per-participant intervention costs were estimated to be €148 (US $176).

### Analysis

#### Statistical Analyses

Analyses were carried out on an intention-to-treat basis. Therefore, all participants were analyzed in the condition to which they were randomized and missing data at post-test for the BSS (n = 21, 8.9%) were imputed using regression imputation as implemented in Stata data analysis and statistical software (StataCorp LP, College Station, Texas, USA) with age, gender, employment status, education, relationship status, nationality, baseline clinical outcomes (ie, suicidal ideation, depression, hopelessness, worry, and anxiety) and randomization status as predictor variables.

For suicidal ideation, a reliable and clinically significant change was calculated to be 6.48 points on the BSS according to the Jacobson and Truax method [46]. Participants were dichotomized according to this criterion into treatment responders and non-responders.

In addition to the primary clinical outcome, the use of the safety procedures is reported (in number of phone calls with participants who exceeded cutoff scores during the trial and referrals to the family physician), as well as the number of suicide attempts.

#### Cost-Effectiveness Analyses

Missing cost data at post-test (between 1% and 18% depending on the type of costs) were imputed using similar regression imputation as for the BSS.

The mean total costs for each of the conditions were calculated at baseline and post-test. Since mean baseline costs were similar across both conditions (see Results), the incremental costs were calculated as the between-group difference at post-test. For reasons of comparability, annualized costs are presented.

Both incremental costs and incremental effects were used to calculate the incremental cost-effectiveness ratio (ICER). The ICER was calculated as (*C*_1 _− *C*_0_)/(*E*_1 _− *E*_0_), where *C *is the average annual per-participant cost and *E *is the proportion of treatment responders in the experimental and control conditions (subscripted 1 and 0, respectively). The ICER describes the incremental costs for gaining one additional treatment response [47-49]. One additional treatment response is defined as 1 participant improving at least 6.48 points on the BSS.

Non-parametric bootstraps were used to simulate 2500 ICERs that were plotted on the cost-effectiveness plane. In this way, the degree of uncertainty associated with the ICER is captured [50]. Each simulated ICER can potentially fall into one of the four quadrants of the ICER plane. The northeast (NE) quadrant represents superior health gains associated with the intervention, but at additional costs relative to routine care. This scenario is typically encountered in economic evaluations—better health is obtained for additional costs. In the northwest (NW) quadrant, health diminishes while costs increase. Clearly, this is the worst possible outcome because the intervention is “dominated” by CAU. In the southwest (SW) quadrant, health diminishes, but there are cost savings. Finally, in the southeast (SE) quadrant, the intervention generates superior health gains (relative to the comparator condition) and does so for lower costs; the intervention “dominates” the comparator condition, which is the best possible outcome.

Use of willingness to pay (WTP) estimates is another method for determining value for money. By assigning hypothetical maximum WTP amounts (ceilings), ranging from €0 to €100,000 per treatment responder, probability estimates for the acceptability of the intervention compared with CAU from a cost-effectiveness point of view, were calculated. The relationship between each assigned WTP ceiling and the probability that the new intervention is viewed as acceptable, can be plotted in an ICER acceptability curve.

#### Sensitivity Analysis

The estimated per-participant intervention costs are surrounded by some uncertainty. To ascertain the robustness of the overall findings, all analyses were repeated for three alternative scenarios, encompassing 10, 20, or 30 minutes of additional guidance per participant, per module (ie, 1, 2, and 3 hours, respectively, per participant during the intervention). These are relevant scenarios because guidance is often provided with Web-based interventions. It was assumed that guidance would be provided by a clinical psychologist, and conservatively, that more therapist time would not increase clinical effectiveness.

## Results

### Sample Characteristics

Participants had a mean age of 40.9 years (SD 13.7). The majority of the sample was female (156/236, 66.1%) and born in the Netherlands (218/232, 94.0%). Approximately half of the sample had completed high school or intermediate vocational training (112/236, 47.5%) and 38.1% (90/236) had completed higher vocational or academic training. A minority was living with a partner (95/236, 40.3%) and had children (87/232, 37.5%). Half of the sample was in paid employment (116/232, 50.0%). Mean score for suicidal ideation was 14.9 at baseline (SD 7.1) There were no significant differences in sociodemographic or clinical characteristics between the intervention and control groups, indicating that randomization had resulted in comparable groups (see Table 3).

**Table 3:** Baseline characteristics of total sample.

Characteristic		Total (n = 236)	Condition		*P*
			Control (n = 120)	Intervention (n = 116)	
**Gender, n (%)**					
	Female	156 (66.1)	80 (66.7)	76 (65.5)	.85
Age, mean (SD)		40.93 (13.71)	41.39 (13.39)	40.46 (14.07)	.60
**Education, n (%)**					
	Lower	19 (8.1)	8 (6.7)	11 (9.5)	
	Intermediate	112 (47.5)	52 (43.3)	60 (51.7)	.36
	Higher	90 (38.1)	51 (42.5)	39 (33.6)	
	Other	15 (6.4)	9 (7.5)	6 (5.2)	
Living with a partner, n (%)		95 (40.3)	54 (45.0)	41 (35.3)	.13
Has children, n (%)^a^		87 (37.5)	50 (42.0)	37 (32.7)	.14
Born in the Netherlands, n (%)^a^		218 (94.0)	111 (93.3)	107 (94.7)	.65
Paid employment, n (%)^a^		116 (50.0)	59 (49.6)	57 (50.4)	.89
Suicidal thoughts, mean (SD)		14.85 (7.08)	14.50 (7.33)	15.20 (6.82)	.44

^a ^Missing: n = 4 (1 in control and 3 in intervention group).

In the 6 weeks prior to baseline, the mean per-participant total costs were €1227 (SD 2364) in the intervention group and €1323 (SD 1891) in the control group, indicating that randomization produced evenly distributed costs across the conditions (*t*_234 _= 0.346, *P *= .73).

### Safety Procedures

As part of the safety procedures, 50 participants were called because they exceeded cutoff scores on suicidal ideation and/or depressive symptoms (31 in the control group and 19 in the intervention group). For 12 of them, the family physician was called (9 in the control group and 3 in the intervention group). Participants were not pulled from the study after being called as part of the safety procedures. Furthermore, 11 participants reported a suicide attempt (7 in the control group and 4 in the intervention group). No suicides occurred during the study.

### Incremental Costs

The average total annualized per-participant costs were calculated to be €13,303 in the intervention group and €18,343 in the control group. The incremental costs were €13,303 − €18,343 = −€5039 (rounded to the nearest Euro) per participant per year (equivalent to a cost savings of US $5941). Table 4 shows the cost components by condition (control and intervention groups) and time (at baseline and post-test). The main difference between the conditions can be observed in costs associated with productivity losses (ie, costs stemming from absenteeism, presenteeism, and domestic help). There was an increase in costs due to absenteeism and domestic work in the control group between baseline and post-test, whereas these costs decreased in the intervention group.

**Table 4:** Cost distribution by condition and time.

Condition		Test time costs^a^	
		Baseline, € (SD)	Post-test, € (SD)
**Care as usual (n = 120)**			
	Direct medical costs	441.61 (1005.70)	558.70 (953.17)
	Medication costs	19.18 (50.60)	21.43 (57.75)
	Direct non-medical costs	142.02 (288.11)	175.86 (282.23)
	Presenteeism^b^	342.63 (818.06)	278.06 (559.30)
	Absenteeism^b^	337.53 (934.96)	392.50 (1135.25)
	Domestic help costs^b^	37.77 (135.47)	69.37 (448.96)
	Intervention costs	NA	NA
	Total costs^c^	1322.98 (1890.93)	1528.56 (1911.82)
** Online self-help (n = 116) **			
	Direct medical costs	459.31 (1541.31)	459.36 (607.32)
	Medication costs	37.55 (84.70)	28.04 (70.84)
	Direct non-medical costs	145.37 (475.21)	142.11 (180.75)
	Presenteeism^b^	276.56 (620.38)	207.22 (453.04)
	Absenteeism^b^	288.13 (1168.70)	251.29 (914.84)
	Domestic help costs^b^	19.83 (54.47)	15.52 (44.04)
	Intervention costs	NA	148.00 (0)
	Total costs^c^	1226.77 (2364.29)	1244.20 (1404.37)

^a ^Mean costs on monthly basis [41] presented in 2009 values.

^b ^Presenteeism, absenteeism, and domestic help all relate to production losses.

^c ^Total costs are the sum of the other cost components. Differences in the totals are due to rounding.

### Incremental Effectiveness

In the intervention group, 35.3% (41/116) met the criteria for clinically significant change, compared with 20.8% (25/120) in the control group. The difference in effectiveness was 0.353 − 0.208 = 0.15 (SE 0.06). This difference was evaluated using a linear probability model while taking into account the clustered data structure (*z *= 2.51, *P *= .01, 95% CI 0.03 0.26).

#### Incremental Cost-Effectiveness

As noted, the incremental costs were −€5039 (negative costs, hence a cost reduction) and the incremental effect was 0.15. Therefore, the mean incremental cost-effectiveness ratio (ICER) was estimated to be −€5039/0.15 = −€34,727 after rounding (a savings of US $41,325) for an additional treatment response. Using the 2500 bootstraps, the median ICER could be estimated as −€31,921 (a savings of US $37,985), essentially conveying the same message.

**Figure 2:**
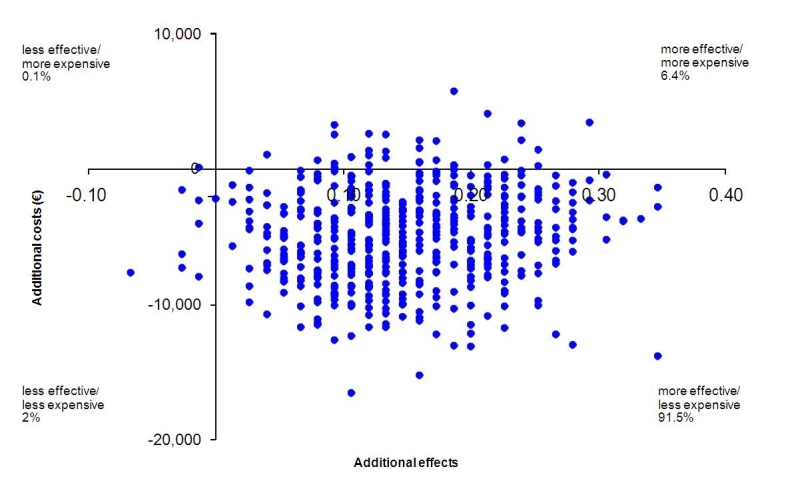
Distribution of bootstrapped incremental cost-effectiveness ratios (ICERs) (n = 2500) on the cost-effectiveness plane, primary analysis.

On the incremental cost-effectiveness plane, each data point represents one simulated ICER. Of these, 91.5% fall into the southeast quadrant, indicating that greater health gains are generated for less cost by the intervention relative to CAU. In addition, 6.4% of the simulated ICERs fall in the northeast quadrant, indicating a probability of 6.4% that by applying the intervention a health gain is produced, but at additional costs. The remainder of the simulated ICERs show up on the west side of the plane, indicating less effectiveness and less cost (2%), or less effectiveness and more cost (0.1%) (see Figure 2).

### Acceptability

**Figure 3:**
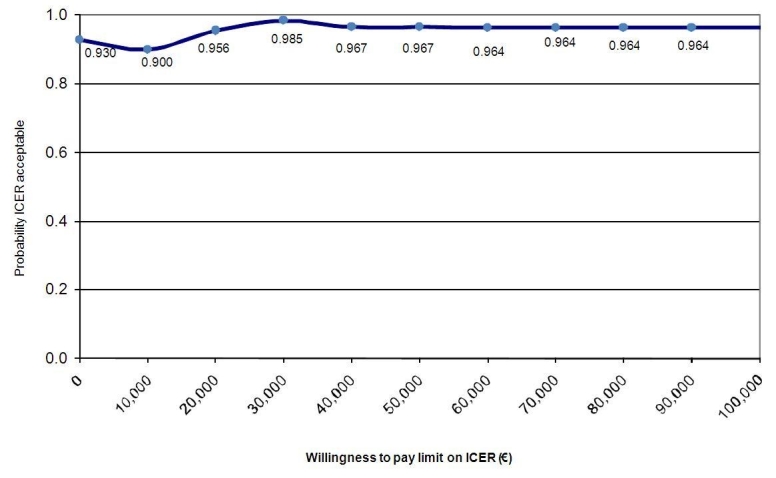
Incremental cost-effectiveness ratio (ICER) acceptability curve: Probability that the intervention is acceptable relative to care as usual (y-axis) given varying thresholds for willingness to pay (x-axis) based on 2500 bootstrap replications.

The incremental cost-effectiveness acceptability curve (Figure 3) suggests that with no willingness to pay for one significantly improved participant, there is a 93% probability that the intervention would be regarded as more cost-effective than CAU. When the willingness to pay for a favorable treatment response is €10,000, €20,000, or €30,000, this probability is 90.4%, 95.6%, and 98.5%, respectively. The minor variations in probabilities between the WTP ceilings imply that the intervention is acceptable from an economic perspective, irrespective of WTP. It can be concluded from this that the intervention can be regarded as acceptable from a cost-effectiveness point of view and that this conclusion is not sensitive to the WTP ceiling used.

### Sensitivity Analysis

Increasing the intervention costs by adding varying amounts of guidance did not affect the overall conclusion that the intervention produces better health outcomes at lower costs, compared with CAU (Table 5). In the first scenario (Scenario A: 1 hour of psychologist support), the median ICER was −€32,342 per treatment responder. The median ICER increased to −€32,708 if the psychologist time was increased by 100%, an ICER that has a 93% probability of falling below the zero willingness to pay threshold (Scenario B: 2 hours of support). Similarly the median ICER increased to −€31,647 if the psychologist time was increased to 180 minutes, an ICER that still has a 93% probability of falling below the zero willingness to pay threshold. The outcomes of these sensitivity analyses are presented in Table 5. These increases in ICERs do not affect the overall conclusion. This indicates that the intervention on top of CAU still produces better health at lower costs, compared with CAU alone.

**Table 5:** Sensitivity analysis of the incremental cost-effectiveness for different scenarios.

Sensitivity analysis		Standard self-help intervention	Scenario		
			A^a^	B^b^	C^c^
Cost, €^d^		−5039	−4900	−4746	−4592
Effect		0.15	0.15	0.15	0.15
incremental cost-effectiveness ratio, median €^e^		−33,593	−32,342	−32,708	−31,647
**Distribution on the cost-effectiveness plane**					
	1^st ^quadrant (northeast)	0.06	0.06	0.06	0.08
	2^nd ^quadrant (inferior: northwest)	0.00	0.00	0.00	0.00
	3^rd ^quadrant (southwest)	0.02	0.01	0.02	0.01
	4^th ^quadrant (dominant: southeast)	0.91	0.92	0.90	0.89
**Willingness to pay ceiling, %**					
	€0	93	93	93	93
	€10,000	90	90	91	88
	€20,000	96	95	96	94
	€30,000	98	99	99	99

^a ^Intervention guided by a clinical psychologist for 10 minutes per module, per participant (ie, 1 hour guidance per participant for the whole intervention).

^b ^Guidance by psychologist for 20 minutes per module, per participant (ie, 2 hours guidance per participant for the whole intervention).

^c ^Guidance by psychologist for 30 minutes per module, per participant (ie, 3 hours guidance per participant for the whole intervention).

^d ^Cost per disease-free year at 2009 prices.

^e ^Median is 50th percentile of 2500 bootstrap replications of the ICER.

## Discussion

### Main Findings

The aim of this paper was to determine whether online self-help for suicidal thoughts would be cost-effective, using data from the first randomized controlled trial comparing online self-help for suicidal ideation on top of CAU to CAU alone. The proportion of participants that showed clinically significant change in suicidal ideation was significantly higher in the intervention group: 35% compared with 21% in the control group. For each significantly improved participant, €34,727 (US $41,325) of societal costs were saved relative to CAU. The finding that different willingness to pay ceilings only minimally affects cost-effectiveness probability estimates also demonstrates that it is a preferable option from a health economic point of view. Sensitivity analyses confirmed the robustness of these findings.

In general, these results add to the observation that Web-based interventions can be favorable from an economic perspective for a range of disorders [8]. However, because no previous cost-effectiveness analyses have been reported for online self-help for suicidal ideation, the obtained results cannot be directly compared. Therefore, it may be more appropriate to compare the results with previous cost-effectiveness studies of face-to-face interventions targeting suicidality, although these are also scarce [26-27]. At best, it can be concluded that the obtained results are in line with the finding that MACT is more effective and cheaper in generating a 1% reduction in the proportion of patients with a self-harm episode than CAU [28]. As MACT is a brief and manual-based treatment, it may be more comparable to self-help than regular face-to-face treatment. Furthermore, a review of therapies for borderline personality disorder (in which suicidality is common) assessed cost-effectiveness in terms of costs per “parasuicide event avoided.” Although results indicated that DBT could potentially be cost-effective, the mixture of results, high levels of uncertainty, and other limitations prevented clear supportive conclusions [29]. It is important to keep in mind that the outcomes in the above comparisons related to suicidal behavior, whereas our study was aimed at suicidal ideation.

Because comparison with previous cost-effectiveness studies targeting suicidality is limited, our results may also be compared with interventions for depression, a common mental disorder in people with suicidal ideation. In this respect, economic evaluations have shown that guided online self-help for depression has a high probability (91%) of being cost-effective compared to CAU [11]. Also, unguided online self-help and therapist-delivered online CBT have been found to be more efficient than CAU [51-52].

### Strengths and Limitations

The findings reported here should be interpreted with caution. Firstly, because of the relatively short time period of 6 weeks, it is unknown how the cost-effectiveness of online self-help is affected after a longer follow-up period. Some economic costs may not have been incurred in this period. Secondly, data on health care consumption and production loss in this study were based on self-report, which may have introduced recall bias. For example, self-report of health care uptake may have been underestimated or overestimated, depending upon the health resource [53-55]. However, because participants were randomized, such a bias is expected to occur in both groups. Finally, several assumptions and estimates were made when calculating the per-participant intervention costs, in particular the number of people who would engage in online self-help for suicidal ideation and costs related to website maintenance. Because these estimates could not be based entirely on actual data, they would need “real world” verification. Unforeseen variations may arise after implementation.

Strengths of this study pertain to its randomized design and the large sample size. Furthermore, because health care utilization data and data on production losses were available, it was possible to study the cost-effectiveness of the intervention from a societal perspective. However, possible spillover effects onto third parties not involved in this study (eg, effects on family and friends) could not be taken into account because data on these were not collected or available.

### Implications

From a research perspective, it is evident that this study needs replication to verify results both within and outside the Netherlands. The latter may be challenging because this would require designing safety procedures that match both general and local ethical and legal considerations, for which no ready-made recipe is available. Moreover, this holds true for implementation of the intervention in practice as well.

From a clinical perspective, it is important to keep in mind that suicidal ideation was the primary focus of this study. It was not designed to detect differences at the level of attempted suicide, so it is unknown whether these could be decreased by online self-help. Evidently, the same is true regarding suicide. However, it still seems a remarkable result that suicidal ideation can be reduced in a cost-effective way, especially given that all participants were encouraged to engage in CAU. Moreover, the control group made more use of this than the intervention group, further strengthening the findings. Similarly, the fact that participants in the control group were called more often due to exceeding the cutoff score supports this.

Finally, it is important to note that this online self-help program was not meant to replace face-to-face contact, but was designed for people who are reluctant to seek face-to-face care. Ideally, people who struggle with suicidality should be seen in person by mental health professionals. In this respect, the intervention studied here can also serve as a complement to face-to-face treatment.

### Conclusion

With respect to psychological interventions targeting suicidality, economic evaluations are practically non-existent. Findings suggest that offering an online intervention on top of CAU increases the likelihood of a favorable outcome. Moreover, these improved clinical outcomes are achieved at lower cost. However, more studies with longer follow-up periods are needed to further substantiate these findings. Therefore, results of this economic evaluation may best be regarded as initial tentative proof of a promising online self-help intervention for suicidal thoughts.

## Multimedia Appendix 1

CONSORT-eHealth Checklist V1.6 [56].

## Abbreviations

BDI: Beck Depression Inventory
BSS: Beck Scale for Suicide Ideation
CAU: care as usual
CBT: cognitive behavior therapy
DBT: dialectical behavior therapy
ICER: incremental cost-effectiveness ratio
MACT: manual-assisted cognitive behavior therapy
MBCT: mindfulness-based cognitive therapy
PPP: purchasing power parity
WTP: willingness to pay
