# Supplementary material for: Reducing Suicidal Ideation: Cost-Effectiveness Analysis of a Randomized Controlled Trial of Unguided Web-Based Self-help
Source: J Med Internet Res. 2012 Oct 26;14(5):e141. doi: 10.2196/jmir.1966 (PMC3517339; doi:10.2196/jmir.1966)
Supplement: Supplementary file 1 [file jmir_v14i5e141_app1.pdf]

CONSORT-EHEALTH Checklist V1.6 Report

Manuscript Number 1966

Date completed 8/25/2011 14:50:29

by Bregje van Spijker

Reducing suicidal ideation via the Internet: Cost-effectiveness analysis alongside a randomized controlled trial into unguided self-help

TITLE

1a-i) Identify the mode of delivery in the title

"web-based"

1a-ii) Non-web-based components or important co-interventions in title

"unguided"

1a-iii) Primary condition or target group in the title

"Reducing suicidal ideation"

ABSTRACT

1b-i) Key features/functionalities/components of the intervention and comparator in the METHODS section of the ABSTRACT

"online unguided self-help intervention for reducing suicidal ideation."
"The intervention aims at decreasing the frequency and intensity of suicidal ideation, consists of six modules and is based on cognitive behavioural techniques."
"a waitlist information-only control group"
"Participants in both groups had unrestricted access to care as usual."

1b-ii) Level of human involvement in the METHODS section of the ABSTRACT

"unguided"

1b-iii) Open vs. closed, web-based (self-assessment) vs. face-to-face assessments in the METHODS section of the ABSTRACT

"All questionnaires were self-report and administered via the internet"

1b-iv) RESULTS section in abstract must contain use data

The ms reports results of cos-effectiveness analyses. Adherence is discussed in greater detail in a clinical results paper.

1b-v) CONCLUSIONS/DISCUSSION in abstract for negative trials

not applicable; our trial was not negative

INTRODUCTION

2a-i) Problem and the type of system/solution

"Suicidal ideation is highly prevalent and causes considerable disease burden, but often remains untreated"
"The rationale for using online-delivery for this intervention included the reach, accessibility, and anonymity of the web, facilitating dissemination"

2a-ii) Scientific background, rationale: What is known about the (type of) system

"The potential economic advantages of web-based interventions are among commonly cited motivations for their development. Indeed, promising results have been published for web-based interventions targeting both somatic and psychological problems. However, being a relatively young research field, economic evaluations of web-based interventions are still scarce and often have limitations"

METHODS

3a) CONSORT

"This paper reports the results of an economic evaluation alongside a randomized controlled trial comparing online self-help for suicidal ideation with a waitlist control condition."

3b-i) Bug fixes, Downtimes, Content Changes

not applicable (did not happen)

4a-i) Computer / Internet literacy

Computer/internet literacy was not assessed directly. However, as the application procedure was web-based, people who are not computer/internet literate would probably not be able to complete the procedure.

4a-ii) Open vs. closed, web-based vs. face-to-face assessments:

"Participants were recruited between October 2009 and November 2010 from the general population by means of advertisements in newspapers, relevant websites (e.g. www.113Online.nl), and Google Adwords."
"After returning the informed consent form, on which participants had to disclose their identity and that of their General Practitioner (GP), ..."

4a-iii) Information giving during recruitment

This is only briefly addressed in the current cost-effectiveness manuscript ("The majority of eligible respondents (n=417, 59.1%) did not return their informed consent form, possibly due to the lack of anonymity when participating"), but is addressed in more detail in a clinical results paper.

4b-i) Report if outcomes were (self-)assessed through online questionnaires

"Questionnaires were self-report and administered via the internet."

4b-ii) Report how institutional affiliations are displayed

NA: no indications that this may have biased results

5-i) Mention names, credential, affiliations of the developers, sponsors, and owners

The content of the intervention was written by B.A.J. van Spijker, A.J.F.M. Kerkhof, and A. van Straten, with the help of an expert panel. The website was built by an external company that had no further role in the study.

5-ii) Describe the history/development process

NA: no previous evaluations are available

5-iii) Revisions and updating

NA: paper describes results of the first trial conducted with the intervention.

5-iv) Quality assurance methods

NA

5-v) Ensure replicability by publishing the source code, and/or providing screenshots/screen-capture video, and/or providing flowcharts of the algorithms used

NA

5-vi) Digital preservation

http://cursus.levenondercontrole.nl

5-vii) Access

Participants accessed the intervention mostly from home. Participation was free of charge.

5-viii) Mode of delivery, features/functionalities/components of the intervention and comparator, and the theoretical framework

The intervention is briefly described in the paper. A more detailed description is available in the protocol paper: van Spijker, BAJ, van Straten, A. & Kerkhof, AJFM. (2010). The effectiveness of a web-based self-help intervention to reduce suicidal thoughts: A randomized controlled trial. Trials 11, 25.

5-ix) Describe use parameters

"Participants were encouraged to follow one module per week and received a weekly automated motivating e-mail. About half (56.0%) of the participants in the intervention group completed at least three modules of the intervention. 21.6% completed the whole intervention, and a similar percentage (22.4%) did not start the intervention."

5-x) Clarify the level of human involvement

"If desired, participants were able to ask questions pertaining to the intervention via the website. These were answered by the researchers, taking an average of 6 minutes per participant over the entire intervention period."

5-xi) Report any prompts/reminders used

"Participants were encouraged to follow one module per week and received a weekly automated motivating e-mail".

5-xii) Describe any co-interventions (incl. training/support)

NA: no co-interventions used.

6a-i) Online questionnaires: describe if they were validated for online use and apply CHERRIES items to describe how the questionnaires were designed/deployed

Questionnaires were not validated for online use

6a-ii) Describe whether and how “use” (including intensity of use/dosage) was defined/measured/monitored

Use was defined as number of modules completed.

6a-iii) Describe whether, how, and when qualitative feedback from participants was obtained

Qualitative feedback was obtained at three month follow-up. This is not included in the current paper, but will be reported in another one.

7a-i) Describe whether and how expected attrition was taken into account when calculating the sample size

"Sample size was based on the expected effect on the primary outcome measure, i.e. the reduction of suicidal thoughts. In order to be able to detect an effect size of 0.35 with α=.05 and β=.80, 100 subjects were needed in each condition. Including an expected drop-out attrition rate of 20-30% in each group, the sample size was determined at 260."

7b) CONSORT

NA

8a) CONSORT

"...,participants (n=236) were stratified for gender and randomized in blocks of size 20 to the intervention (n=116) or the waitlisted information control condition (n=120) by an independent researcher using random allocation software."

8b) CONSORT

see 8a

9) CONSORT

see 8a

10) CONSORT

see 8a.
"Eligibility was assessed using an online application procedure"

11a-i) Specify who was blinded, and who wasn't

NA

11a-ii) Discuss e.g., whether participants knew which intervention was the "intervention of interest" and which one was the "comparator"

Participants were informed about this.

11b) CONSORT

NA

12a) CONSORT

"Statistical analyses
Analyses were carried out on an intention-to-treat basis. Therefore, all participants were analyzed in the condition to which they were randomized and missing data at post-test for the BSS (n=21, 8.9%) were imputed using regression imputation as implemented in Stata, with age, gender, employment status, education, relationship status, nationality, baseline clinical outcomes (suicidal ideation, depression, hopelessness, worry, anxiety) and randomization status as predictor variables.
For suicidal ideation, reliable and clinically significant change was calculated to be 6.48 points on the BSS according to the Jacobson and Truax method [43]. Participants were dichotomized according to this criterion into treatment responders and non-responders.
In addition to the primary clinical outcome, the use of the safety procedures is reported (in number of phone calls with participants who exceeded cutoff scores during the trial and referrals to GP), as well as number of suicide attempts.

Cost-effectiveness analyses
Missing cost data at post-test (between 1% and 18% depending on the type of costs) were imputed using similar regression imputation as for the BSS. The mean total costs for each of the conditions were calculated at baseline and post-test. Since mean baseline costs were similar across both conditions (see Results), the incremental costs were calculated as the between-group difference at post-test. For reasons of comparability, annualized costs are presented.
Both incremental costs and incremental effects were used to calculate the incremental cost-effectiveness ratio (ICER). The ICER was calculated as (C1-C0)/(E1-E0), where C is the average annual per participant cost and E is the proportion of treatment responders in the experimental and control conditions (subscripted 1 and 0 respectively). The ICER describes the incremental costs for gaining one additional treatment response [44-46]. One additional treatment response is defined as one participant improving at least 6.48 points on the BSS.
Non-parametric bootstraps were used to simulate 2,500 ICERs that were plotted on the cost-effectiveness plane. In this way, the degree of uncertainty associated with the ICER is captured [47]. Each simulated ICER can potentially fall into one of the four quadrants of the ICER plane. The North-East (NE) quadrant represents superior health gains associated with the intervention, but at additional costs relative to routine care. This scenario is typically encountered in economic evaluations: better health is obtained for additional costs. In the North-West (NW) quadrant health diminishes while costs increase. Clearly, this is the worst possible outcome, as the intervention is “dominated” by CAU. In the South-West (SW) quadrant health diminishes, but there are cost-savings. Finally, in the South-East (SE) quadrant the intervention generates superior health gains (relative to the comparator condition) and does so for lower costs; the intervention “dominates” the comparator condition, which is the best possible outcome.
Use of willingness to pay (WTP) estimates is another method for determining value for money. By assigning hypothetical maximum WTP amounts (ceilings), ranging from €0 to €100,000 per treatment responder, probability estimates for the acceptability of the intervention compared with CAU from a cost-effectiveness point of view, were calculated. The relationship between each assigned WTP ceiling and the probability that the new intervention is viewed as acceptable, can be plotted in an ICER acceptability curve.

Sensitivity analysis
The estimated per-participant intervention costs are surrounded by some uncertainty. To ascertain the robustness of the overall findings, all analyses were repeated for three alternative scenarios, encompassing 10, 20, or 30 minutes of additional guidance per participant, per module (i.e. 1, 2, and 3 hours, respectively, per participant during the intervention). These are relevant scenarios as guidance is often provided with web-based interventions. It was assumed that guidance would be provided by a clinical psychologist, and, conservatively, that more therapist time would not increase clinical effectiveness."

12a-i) Imputation techniques to deal with attrition / missing values

see 12a

12b) CONSORT

see 12a

RESULTS

13a) CONSORT

see figure 1 in paper

13b) CONSORT

see figure 1 in paper

13b-i) Attrition diagram

Attition is described in more detail in a paper on the clinical effectiveness, which is currently under review with the British Journal of Psychiatry.

14a) CONSORT

"Participants were recruited between October 2009 and November 2010"

14a-i) Indicate if critical “secular events” fell into the study period

NA: no secular events happened
14b) CONSORT
NA
15) CONSORT
see table 3.
15-i) Report demographics associated with digital divide issues
see table 3.
16-i) Report multiple “denominators” and provide definitions
see figure 1
16-ii) Primary analysis should be intent-to-treat
"Analyses were carried out on an intention-to-treat basis."
17a) CONSORT
"Incremental costs
The average total annualized per-participant costs were calculated to be €13,303 in the intervention group and €18,343 in the control group. The incremental costs were therefore €13,303 - €18,343 = - €5,039 (rounded to the nearest Euro) per participant per year (equivalent to a cost saving of US$5,941). Table 4 shows the cost components by condition (control and intervention group) and time (at baseline and posttest). The main difference between the conditions can be observed in costs associated with productivity losses, i.e. costs stemming from absenteeism, presenteeism and domestic help. There was an increase in costs due to absenteeism and domestic work in the control group between baseline and posttest, while these costs decreased in the intervention group.

Incremental effectiveness
In the intervention group 35.3% (41/116) met criteria for clinically significant change, compared with 20.8% (25/120) in the control group. The difference in effectiveness was therefore 0.353 – 0.208 = 0.15 (SE 0.06). This difference was evaluated using a linear probability model while taking into account the clustered data structure (z=2.51, P=.01, 95% CI 0.03 - 0.26).

Incremental cost-effectiveness
As noted, the incremental costs were - €5,039 (negative costs, hence a cost reduction) and the incremental effect was 0.15. Therefore, the mean incremental cost-effectiveness ratio (ICER) was estimated to be - €5,039 / 0.15 = - €34,727 after rounding (a saving of US$41,325) for an additional treatment response. Using the 2,500 bootstraps, the median ICER and its 95% confidence interval could be estimated as - €31,921 (a saving of US$37,985), essentially conveying the same message.
On the incremental cost-effectiveness plane, each data point represents one simulated ICER. Of these, 91.5% fall into the SE-quadrant, indicating that greater health gains are generated for less cost by the intervention relative to CAU. In addition, 6.4% of the simulated ICERs fall in the NE-quadrant, indicating a probability of 6.4% that by applying the intervention a health gain is produced, but at additional costs. The remainder of the simulated ICERs show up on the west side of the plane, indicating less effectiveness and less cost (2%), or less effectiveness and more cost (0.1%) (see Figure 2)."
17a-i) Presentation of process outcomes such as metrics of use and intensity of use
"In estimating per-participant intervention costs, the average time spent on the intervention was valued at €12.50 per hour (leisure time value) for an average of 10.5 hours per participant over the 6-week intervention period."
17b) CONSORT
see 17a
18) CONSORT
NA
18-i) Subgroup analysis of comparing only users
NA
19) CONSORT
"Safety procedures
As part of the safety procedures, 50 participants were called because they exceeded cutoff scores on suicidal ideation and/or depressive symptoms (31 in control and 19 in intervention group). For 12 of them the GP was called (9 in control and 3 in intervention group). Furthermore, eleven participants reported a suicide attempt, of whom seven were in the control group. No suicides occurred during the study."
19-i) Include privacy breaches, technical problems
NA

19-ii) Include qualitative feedback from participants or observations from staff/researchers

NA. (not included in the current cost-effectiveness paper, but in the clinical papers)

DISCUSSION

20-i) Typical limitations in ehealth trials

"The findings reported here should be interpreted with caution. Firstly, due to the relatively short time-horizon of 6 weeks it is unknown how the cost-effectiveness of online self-help is affected after a longer follow-up period. Secondly, data on healthcare consumption and production loss in this study were based on self-report, which may have introduced recall bias. For example, self-report of healthcare uptake may have been underestimated or overestimated, depending upon health resource [50-52]. However, as participants were randomized, such a bias is expected to occur in both groups. Finally, several assumptions and estimates were made when calculating the per-participant intervention costs, in particular the number of people that would engage in online self-help for suicidal ideation and costs related to website maintenance. As these estimates could not entirely be based on actual data, they would need 'real-world' verification. Unforeseen variations may therefore arise after implementation. "

21-i) Generalizability to other populations

"From a clinical perspective, it is important to keep in mind that suicidal ideation was the primary focus of this study. It was not powered to detect differences at the level of attempted suicide, so it is unknown whether these could be decreased by online self-help. Evidently, the same is true regarding suicide. "

21-ii) Discuss if there were elements in the RCT that would be different in a routine application setting

NA. This is described in the clinical papers that are currently under review, not in the cost-effectiveness paper.

22-i) Restate study questions and summarize the answers suggested by the data, starting with primary outcomes and process outcomes (use)

"Main findings

The aim of this paper was to determine whether online self-help for suicidal thoughts would be cost-effective, using data from the first randomized controlled trial comparing online self-help for suicidal ideation on top of CAU with CAU alone. The proportion of participants that showed clinically significant change in suicidal ideation was significantly higher in the intervention group: 35% compared with 21% in the control group. For each significantly improved participant, €34,727 (US$41,325) of societal costs were saved relative to CAU. The finding that different willingness to pay ceilings only minimally affect cost-effectiveness probability estimates also demonstrates that it is a preferable option from a health economic point of view. Sensitivity analyses confirmed the robustness of these findings. "

22-ii) Highlight unanswered new questions, suggest future research

"From a research perspective, it is evident that this study needs replication to verify results both within and outside the Netherlands. "

Other information

23) CONSORT

"Netherlands Trial Register, NTR1689 http://www.trialregister.nl/trialreg/admin/rctview.asp?TC=1689 "

24) CONSORT

"The methods used in this trial have been described in detail elsewhere (van Spijker, B. A. J., van Straten, A., & Kerkhof, A. J. F. M. (2010). The effectiveness of a web-based self-help intervention to reduce suicidal thoughts: A randomized controlled trial. Trials 11, 25.)

25) CONSORT

"The study was supported by a grant from the Netherlands Health Research Council, The Hague (ZonMw) (project number 120510003). This funding organization was not involved in the design and conduct of the study; the collection, management, analysis, and interpretation of the data; and the preparation, review, or approval of the manuscript. "

X26-i) Comment on ethics committee approval

NA

x26-ii) Outline informed consent procedures

"After returning the informed consent form, on which participants had to disclose their identity and that of their General Practitioner (GP),..."

X26-iii) Safety and security procedures

"Because this study was conducted in a vulnerable population, safety procedures were employed. Each time a participant in either condition exceeded cut-off scores on suicidal ideation or depressive symptoms, a risk assessment was carried out over the phone. If necessary, or if a participant could not be reached, their GP was contacted."

X27-i) State the relation of the study team towards the system being evaluated

"The authors declare that they have no conflict of interest. "
